# Correction: HaptiKart: An engaging videogame reveals elevated proprioceptive bias in individuals with autism spectrum disorder

**DOI:** 10.1371/journal.pdig.0001482

**Published:** 2026-06-01

**Authors:** Daniel E. Lidstone, Mohit Singhala, Liam J. Wang, Jeremy D. Brown, Stewart H. Mostofsky

The following information is missing from the Funding statement: IDDRC grant number (P50 HD103538; Schlaggar).
